# Beyond the Check Box: Development of the Nutrition Health Related Social Need Assessment and Referral Tool (N-HART)

**DOI:** 10.1007/s11606-025-10048-0

**Published:** 2025-12-02

**Authors:** Kristin L. Rising, Mackenzie Kemp, Angela Gerolamo, Nina Diamond, Paula Ostroff, Sara Malone, Karen Alexander, Rhea Powell

**Affiliations:** 1 Center for Connected Care, Thomas Jefferson University, Philadelphia, PA USA; 2 Department of Emergency Medicine, Sidney Kimmel Medical College, Thomas Jefferson University, Philadelphia, PA USA; 3 Department of Surgery, Washington University School of Medicine in St. Louis, St. Louis, MO USA; 4 Friends Research Institute, Philadelphia, PA USA; 5 Department of Internal Medicine, Sidney Kimmel Medical College, Thomas Jefferson University, Philadelphia, PA USA

**Keywords:** social determinants of health, nutrition assessment, food insecurity, access to healthy food

## Abstract

**Background:**

Food insecurity screeners focus on identifying a financial barrier to accessing food. Other social and medical factors influence an individual’s nutritional status and the need for food provision support. Yet, there are no existing tools that elicit the various life factors that influence food and nutrition needs and further identify relevant resources to address those needs.

**Objective:**

To develop the Nutrition Health Related Social Need Assessment and Referral Tool (N-HART) to facilitate individualized referrals to food and nutrition resources, based on both medical and social factors. Resources include food provision, nutrition and cooking education, and benefits assistance.

**Design:**

We used a multi-phase approach to develop and refine the N-HART from March 2023 to March 2024. An initial draft of the N-HART was developed based on a literature review and field scan. Cognitive interviews with patients were used to iteratively refine the tool.

**Participants:**

The tool was developed for use across a health system, applicable to both inpatient and ambulatory settings. We conducted cognitive interviews with 15 patients at an acute care hospital in Philadelphia, PA to inform the refinement of the N-HART.

**Key Results:**

The N-HART is a 13-item dual-function tool that screens for food and nutrition needs and recommends a best fit food resource type (e.g., medically tailored meals, non-tailored prepared meals, food pantry) based on an individual’s needs. Cognitive interviews informed changes in N-HART framing and period of focus, and led to the elimination of some items to be as efficient and actionable as possible.

**Conclusions:**

With health systems focusing on HRSN, the N-HART may provide a strategy for informing more individualized nutrition referrals using a structured approach.

**Supplementary Information:**

The online version contains supplementary material available at 10.1007/s11606-025-10048-0.

## BACKGROUND

Hunger and nutrition-related health crises continue to rise in the USA, from 11% in 2018 to 13.5% in 2023.^1,2^ Food insecurity is associated with myriad adverse health impacts, including increased risk of chronic diseases, lower diet quality, cognitive problems, and poorer mental health.^3,4^ Health system wide food insecurity screening has become more common over the past decade due to advocacy from professional medical associations,^5,6^ payment demonstrations,^7^ and regulatory mandates.^8^ Yet research suggests that screening solely for financial barriers may miss patients needing food resources due to other life factors.^9,10^ For example, a patient undergoing cancer treatment may be able to afford food yet be unable to prepare meals due to debilitating fatigue, putting them at increased nutritional risk.

In addition to limitations of current screeners to capture the full scope of food-related need, health systems and providers lack guidance and tools to efficiently and effectively address needs once they are identified. System barriers such as limited time and capacity, insufficient knowledge of community resources, unclear workflows, and provider burnout contribute to patients’ social needs going unaddressed.^11–13^ As such, there is a critical need to facilitate health systems’ abilities to refer patients to the appropriate tier of food resources and services.^14^ A recent study called for research to evaluate how to best connect patients with appropriate resources, close the loop on whether assistance is actually provided, and which forms of assistance are most helpful for addressing health related social needs (HRSN).^15^ There are no existing tools that both elicit functional life factors that contribute to food and nutrition needs and provide optimal referrals.

To address this critical gap, we developed the Nutrition HSRN Assessment and Referral Tool (N-HART) to facilitate individualized referrals for people facing various levels of food and nutrition challenges. The N-HART takes medical and social situations into consideration to produce resource referrals that are tailored to an individual’s specific food needs. It is designed to be used across adult healthcare or community settings, and can be used as a standalone tool or as a supplement to standard HRSN screening. In the following, we describe the development and refinement of the N-HART.

## METHODS

### Study Design and Setting

We used a multi-stage approach to develop and refine the N-HART from March 2023 to March 2024. This approach included (1) a literature review of existing validated food insecurity and malnutrition screening tools; (2) a field scan of food resources in the Philadelphia area, including discussions with leaders of community-based organizations; (3) the development of a draft N-HART; and (4) the refinement of the N-HART through cognitive interviews with patients.

The setting was an acute care hospital in Philadelphia, PA. Participants provided verbal consent to be interviewed. Study activities were approved by the university’s Institutional Review Board.

### Sample/Participants

Patients were eligible to participate in a cognitive interview if they were 18 years or older; were English-speaking; were hospitalized; resided in Philadelphia, PA; and either screened positive for at least one HRSN or were identified by hospital staff as having a nutrition need. Exclusion criteria included being unhoused, being physically unable to feed oneself, having cognitive impairment, or being institutionalized. These individuals were excluded either because their current status did not allow for receipt of most food services or because their cognitive status would not allow for full participation in required study activities. Recruitment entailed reviewing the electronic medical record (EMR) daily to identify potentially eligible patients, and then approaching them to confirm eligibility and ascertain study interest.

### Data Collection

#### Literature Review

The first step in N-HART development was a literature review of published validated assessment tools that assess food insecurity or malnutrition. The team searched PubMed for peer-reviewed articles on the psychometric properties of food insecurity, malnutrition, or health-related social needs screening tools, as well as relevant systematic reviews. Search terms included validation, psychometric, food insecurity screener, malnutrition screener, health related social needs screener, and social risk screener. We also asked hospital social workers and dietitians to provide assessment tools currently being used within the health system. Exemplar or most relevant assessment tools were selected by consensus among the study team. The team reviewed all questions within each tool and categorized each question by barrier, target population, and administrative features. A question-level matrix was constructed to facilitate exploration of item content across tools.

#### Field Scan

Following the literature review, we performed a field scan of local community-based organizations (CBOs) that provide food and nutrition resources in Philadelphia. Field scans, otherwise known as informal landscape analyses, are used to get a broad view of a field to identify key players, gaps, and resources. Scans incorporate various data collection methods, including one-on-one conversations, surveys, and expert input.^16^ We first used the EMR-integrated FindHelp platform to understand the availability of local services.^17^ With consultation from social workers and case managers, we identified nine frequently utilized CBOs that provide services across eight food and nutrition resource types: medically tailored meals (MTM), non-tailored prepared meals, food boxes, food vouchers, food pantries, congregate meal sites, SNAP benefit support, and nutrition education. Nine CBOs were invited for an informal conversation with the research team and we met with six. Conversations explored eligibility requirements, descriptions of services, success metrics, clients for whom they perceived services to be most useful, and referral sources and workflows. Meeting notes were reviewed during team meetings. Data from the field scan were used to confirm the availability of local resource types, identify key service eligibility criteria, and ascertain additional patient factors that were not reflected in existing tools.

#### N-HART Development

An initial N-HART tool was drafted based on the literature review and field scan. We aimed to develop a list of yes/no questions that collectively map to a specific resource type to meet individual nutrition needs (e.g., cancer patient who is too sick to cook to MTM; an individual lacking mobility yet able to cook to home-delivered groceries). The N-HART produces two levels of output: food provision resource outputs and supplemental resource outputs (e.g., benefit assistance, nutrition education). The tool identifies best fit and alternative resources based on assessment responses. All questions were reviewed for simplicity and to ensure a sixth grade reading level using the readability feature in Microsoft Word.^18^

We also gathered expert input from dietitians, social workers, community health workers, physicians, and nurses through individual interviews. Experts were identified by departmental leadership. Input included professionals’ perceptions of food and nutrition needs among hospitalized patients, feasible solutions to adequately identify patients at nutrition risk, and procedures to ensure appropriate referral to nutrition resources.

#### N-HART Refinement

Upon completion of the draft N-HART, we conducted cognitive interviews with inpatients to gain insight regarding participants’ understanding of survey questions. This methodology allows researchers to gain actionable feedback from participants on the clarity, comprehension, and usability of the tool.^19^ Interviews were conducted between January 2024 and March 2024 by two trained interviewers. Interview length ranged from 10 to 50 min. Participants were first asked to answer the N-HART questions. They were then asked to assess each item’s understandability and the information they needed to recall to answer the item. Participants were also asked if any questions should be removed from or added to the tool, and overall feedback on completing the assessment. Participants were informed of the resource outputs suggested by the N-HART and were asked for their opinion on the appropriateness of the referral. The team then placed referrals and followed up with individuals to ensure receipt of services.

Interview notes were documented in real time and conversations were recorded. Audio files were stored on a HIPAA-compliant online platform for review by the study team. The tool was modified iteratively based on interview notes, and interviews were stopped once no further edits were needed. Demographic information was gathered at the time of the interview. Participants were compensated $40 for participation.

## RESULTS

### N-HART Development

The literature review identified four validated food insecurity screeners^20–23^ and five malnutrition screeners.^24–28^ A majority of HRSN assessments did not include unique food questions beyond what was already reflected in the validated food insecurity screeners. See Appendix 1 for a full list. Based on the review of these tools and findings from the field scan, the team identified four food barrier concepts for inclusion in the N-HART: access to needed kitchen equipment, ability to cook or prepare food, ability to shop for food, and ability to afford food. We identified five categories of food provision resource types and three supplemental nutrition services. Food provision resources included home-delivered MTM; home-delivered prepared meals; home-delivered food boxes or groceries; food pantries; and congregate meal sites. Services included free, self-pay, and insurance-covered options. Supplemental nutrition services included enrollment in the Supplemental Nutrition Assistance Program (SNAP) and Supplemental Nutrition Program for Women, Infants and Children (WIC); cooking education; and nutrition education programs. Referral categories reflected the resources available in Philadelphia, so are not exhaustive of all possible resource types.

The initial draft N-HART included four main questions to cover the four food barrier concepts, follow-up questions on specific facilitators and barriers, and an indication of medical factors that may impact nutrition status. The draft tool was reviewed with 24 providers and content experts at the study site. All participants endorsed that the N-HART would add value for facilitating more strategic and data informed referrals, reduce the complexity of understanding what resources may be available for patients, and provide a more complete picture of food and nutrition needs beyond existing HRSN assessments. A potential concern about the N-HART included limited staff capacity to conduct the screening and initiate the referral.

### N-HART Refinement

We engaged 15 participants in cognitive interviews to refine the N-HART. Participants were primarily Black/African American (60%), female (80%), and ranged in age from 34 to 89. Most had a yearly household income of less than $24,999 (60%) and were on Medicaid insurance (73%) (Table 1). Seventy-three percent of the participants screened positive for food insecurity using the Hunger Vital Sign,^21^ while 93% were in need of food resources as identified by the N-HART.

**Table 1:** Demographics of Cognitive Interview Participants (*N* = 15)

Characteristic	*n* (%)
Age – Mean (range)	54.3 (34–89)
Race*	
Black/African American	9 (60.0)
Hispanic or Latino	2 (13.3)
White	4 (26.7)
Gender	
Male	2 (13.3)
Female	12 (80.0)
Decline	1 (1.1)
Highest level of completed education	
Less than high school	4 (26.7)
High school graduate/GED/Some college	10 (66.7)
College degree	1 (6.7)
Current employment status*	
Working full time	5 (33.3)
Working part-time	1 (6.7)
Looking for work/Unemployed	2 (13.3)
Disabled/On Disability	7 (46.7)
Retired	2 (13.3)
Yearly household income	
< $25,000	9 (60.0)
$25,000–49,999	1 (6.7)
$50,000–74,999	1 (6.7)
Unsure/Decline	4 (26.6)
Insurance*	
Private	3 (20.0)
Medicare	4 (26.7)
Medicaid	11 (73.3)

^*^Question was select all that apply

Overall, cognitive interview participants had no recommendations for changes in N-HART question wording. They reported that the N-HART was acceptable and useful for understanding their needs and informing referrals to resources. Despite the absence of patient edits to the N-HART, the research team made several adjustments based on information shared by participants during the cognitive interviews. The initial version of the N-HART used strengths-based language to assess participants’ ability to meet their nutrition needs (e.g., “I am able to…”). Interviewers observed that such language did not fully elicit the challenges individuals faced at home, making accurate resource referrals difficult. For example, several patients reported being able to get the food they needed due to family support. However, they felt badly about placing this burden on their support network. As a result, the research team changed the overall language of the tool to focus on identifying barriers (i.e., “do you think you will have problems…”). We specifically modified the wording of three key questions about affording food, shopping for food, and cooking food to reflect this change in perspective. In addition, as many participants discussed anticipated changes in their situation due to current health challenges, we changed the time reference from “in the past month” to “in the next month”.

When asked what information they needed to recall when answering N-HART questions, many participants provided context to the individual factors that influenced their nutrition needs (Appendix 2), which helped confirm that the N-HART was assessing the right concepts. While unintentional weight loss, mechanical difficulties with eating, transportation barriers, and medical limitations provide helpful nuance in understanding a patient’s situation, these questions did not ultimately impact the referral recommendation and were removed from the N-HART. For example, if a participant endorsed having problems with getting food to their home, the N-HART would suggest a home-delivered intervention regardless of whether their specific barrier was due to transportation or medical limitations. By ensuring every question impacted the referral recommendation produced by the N-HART, the team focused on creating the most efficient yet individualized tool possible.

Based on the information captured in the N-HART, the tool maps to an individualized food provision resource category and provides information for specific services available in the community. While the tool emphasizes the best fit resource type, it also provides alternative resource types if individuals turn down the best fit or are ineligible for unforeseen circumstances. Supplemental resources for benefits assistance and education may be suggested for those who indicated interest.

The research team member reviewed the N-HART best fit food provision resource type with the participant and explored the perceived acceptability of that intervention. Upon patient acceptance of the food provision resource category, the team member reviewed available services and made referrals for 11 of the 15 interview participants. Three participants were already receiving a food provision intervention and were not in need of our support and one participant had no food provision need.

The refined N-HART is a 13-item tool that produces individualized referral recommendations to food provision and supplemental resources for people with nutrition needs (Appendix 3). The primary utility of the N-HART is to assess an individual’s ability to store and heat food, prepare food, physically get food into the home, and afford food. It also considers medical conditions and basic demographic information relevant to resource eligibility, and incorporates interest in nutrition and cooking education. Based on the information captured in the N-HART, the tool maps to an individualized food provision resource category and provides information for specific services available in the community.

## DISCUSSION

We developed and refined the N-HART with significant patient and stakeholder input as a tool to support the referral of individuals to a best fit category of food and nutrition resources. We identified multiple questions that are important to inform patients’ nutrition needs that go beyond the sole assessment of financial constraints, highlighting the importance of a tool such as the N-HART to identify individuals who need assistance and to inform the most effective referrals. Further, we found that the N-HART was acceptable to patients and was useful for informing food and nutrition referrals.

Food is medicine interventions have shown improvements in disease management and health status and help narrow gaps in food insecurity and health disparities.^29–35^ Federal and state efforts focused on expanding nutrition-related coverage offer promise for more widespread integration of food is medicine approaches in the healthcare setting, such as coverage of MTM. The N-HART can fill research gaps by streamlining the process to determine intervention dose (resource category) and duration,^36^ as well as identifying structural factors (e.g., lack of working kitchen equipment) that need to be addressed to optimize referral impact.

This work has far-reaching implications for healthcare organizations, insurance companies, and policymakers. As health systems have shifted toward widespread screening for HRSN, there is a significant opportunity to intervene on key factors contributing to persistent health inequities. Research suggests that engaging in screening as a task will not be successful without integrating it into clinical decision-making and ensuring that patients view those who are engaged in referrals and follow-up as part of their healthcare team.^37^ Furthermore, the N-HART has the potential to create a more efficient approach to nutrition need identification and referral navigation for hospital staff (e.g., social workers, community health workers) that are already working at or beyond capacity.

The N-HART may also address important and persistent barriers to effectively addressing HRSN as identified in a comprehensive evaluation of the Accountable Health Communities model, which found that maintaining a community resource inventory and being aware of changes to program availability and eligibility were labor and time intensive.^38,39^ The N-HART has the potential to support resource navigation staff in identifying, at a minimum, the most relevant resource type based on an individual’s needs, reducing time spent exploring resource options.

An important consideration for implementation is that the N-HART requires adaptations to incorporate locally available services and their eligibility criteria. While this will require an upfront time investment, it will likely save time in the long term. In subsequent work, we found multiple insurance-based nutrition offerings of which both patients and health system providers were largely unaware. While the N-HART does not incorporate insurance as a variable to determine a best fit resource type, coverage should be used to identify services within that resource type a patient may be eligible for.

This work has limitations. Development of the N-HART was informed by data collected from a single urban tertiary care hospital in Philadelphia, PA. While concepts covered within the N-HART are not unique to Philadelphia, testing is needed in other settings to support generalizability. In addition, the N-HART is most useful when paired with a thorough and dynamic guide of local resources. As such, use of the N-HART in settings outside Philadelphia, PA will require investment by other teams to develop the needed resource lists to support tiered referrals. Resource guides should be updated as the service landscape changes. Finally, this work does not report on actual testing of the N-HART in the clinical environment, as that work is currently underway.

## CONCLUSION

The N-HART was developed as a strategy to incorporate the complex medical and social factors that influence an individual’s nutrition needs into individualized resource referrals. Refinement and early testing with patients demonstrate that the N-HART holds promise as an acceptable and useful tool for informing nutrition referrals among patients in the acute care setting. Using the N-HART to address nutrition needs may lead to partial or complete food insecurity resolution and improved health outcomes. Our team is currently conducting an implementation trial of the N-HART in the acute care setting in which we will collect preliminary data on patient-level outcomes as well as implementation outcomes to assess N-HART screening and referral acceptability, appropriateness, and uptake. The trial will also inform the resources required, barriers, and facilitators to operationalizing approaches to addressing nutrition insecurity in the acute care setting. Future work will look to integrate the N-HART into existing clinical HRSN workflows, test the use of the N-HART in outpatient settings and expand the N-HART approach to other HRSN domains.

## Supplementary Information

Below is the link to the electronic supplementary material.

Supplementary Material 1 (DOCX 16.0 KB)

Supplementary Material 2 (DOCX 16.3 KB)   

Supplementary Material 3 (DOCX 19.4 KB)
