# Supplementary material for: Beyond the Check Box: Development of the Nutrition Health Related Social Need Assessment and Referral Tool (N-HART)
Source: J Gen Intern Med. 2025 Dec 2;41(8):2073–9. doi: 10.1007/s11606-025-10048-0 (PMC13241548; doi:10.1007/s11606-025-10048-0)
Supplement: Supplementary file 1 — Supplementary Material 1 (DOCX 16.0 KB) [file 11606_2025_10048_MOESM1_ESM.docx]

**Appendix 1. Validated Tools Included in Literature Review**

| **Validated Tool Name** | **Brief Description of Tool Purpose** |
| --- | --- |
| *Food Insecurity* | |
| USDA U.S. Household Food Security Survey Module | This survey uses a three-stage design with screeners. There are 10 items on the screener that measure an adult’s food security status, ranging from high food security to very low food security among adults. |
| Hunger Vital Sign | A brief screener based on the U.S. Household Food Security Survey Module that identifies households at risk or food insecurity. |
| Single-Question Hunger Screen | One item screening tool to identify family hunger validated in an inner-city pediatric primary health care setting. |
| SEEK One-Item Food Insecurity | Single item food insecurity screener validated in pediatric setting. |
| *Malnutrition* |  |
| Scored Patient-Generated Subjective Global Assessment (PG-SGA) | A validated tool used in oncology and other chronic catabolic conditions to measure weight loss, food intake, symptoms, activities, disease, metabolic demand, and physical exam to stage nutritional risk. |
| Nutrition Risk Screening 2002 (NRS-2002) | A validated tool, endorsed by the European Society for Clinical Nutrition and Metabolism, that aims to screen patients at nutrition risk in the hospital setting. |
| Mini Nutritional Assessment Short Form (MNA-SF) | A nutritional screening tool for the free-living and clinically relevant elderly population that measures nutritional and health conditions, independence, quality of life, cognition, mobility, and subjective health. |
| Malnutrition Screening Tool (MST) | A two-question screener that uses appetite and unintentional weight loss to determine whether an individual is at risk for malnutrition. |
| Malnutrition Universal Screening Tool (MUST) | Developed by the British Association for Parenteral and Enteral Nutrition to detect malnutrition based on nutrition status and disease-related dysfunction |
| *Social Risk* | |
| No relevant validated tools identified. Most social risk assessments incorporated the food insecurity screening questions mentioned above. | |
