# Supplementary material for: Beyond the Check Box: Development of the Nutrition Health Related Social Need Assessment and Referral Tool (N-HART)
Source: J Gen Intern Med. 2025 Dec 2;41(8):2073–9. doi: 10.1007/s11606-025-10048-0 (PMC13241548; doi:10.1007/s11606-025-10048-0)
Supplement: Supplementary file 2 — Supplementary Material 2 (DOCX 16.3 KB) [file 11606_2025_10048_MOESM2_ESM.docx]

**Appendix 2. Individual Factors Influencing Nutrition Needs Based on Feedback Provided During Cognitive Interviews**

| **Individual Factor** | **Participant Experiences** |
| --- | --- |
| Physical limitations to cooking and shopping | - Difficulty standing - Feeling fatigued after cooking or shopping |
| Social limitations to cooking and shopping | - Mental health challenges - Insufficient childcare - Not wanting to burden support networks when help was needed |
| Environmental limitations to cooking and shopping | - Limited or no kitchen space - Broken or insufficient appliances - Relying on public transportation - Distance to culturally preferred stores |
| Financial limitations | - Food is too expensive - Loss of income due to medical conditions - Insufficient Supplemental Nutrition Assistance Program funds - Difficulty navigating benefits system |
