# Supplementary material for: Beyond the Check Box: Development of the Nutrition Health Related Social Need Assessment and Referral Tool (N-HART)
Source: J Gen Intern Med. 2025 Dec 2;41(8):2073–9. doi: 10.1007/s11606-025-10048-0 (PMC13241548; doi:10.1007/s11606-025-10048-0)
Supplement: Supplementary file 3 — Supplementary Material 3 (DOCX 19.4 KB) [file 11606_2025_10048_MOESM3_ESM.docx]

**Appendix 3. Nutrition HRSN Assessment and Referral Tool (N-HART)**

1. Are you interested in being referred for food-related resources if you qualify? – Y/N
   1. **IF NO 🡪 STOP**
2. Do you have a working refrigerator or freezer at home? – Y/N
3. Do you have a way to heat up your food at home? – Y/N
   1. **IF NO TO EITHER 2 or 3** 🡪 **congregate meal site referral**
4. Do you think you will have problems preparing or cooking the food you need for your health in the next month? – Y/N
   1. **IF NO 🡪 skip to question #7**
5. (ONLY ASK IF YES to #4) Do you have any of the following medical conditions?
   1. Diabetes
   2. cancer (on active treatment)
   3. heart disease
   4. HIV/AIDS
   5. kidney disease
   6. hepatitis C/liver disease
      - **IF NO - home-delivered meals (not medically-tailored)**

** Paid, low cost, and free options are needed to meet financial ability

1. (ONLY ASK IF YES to #5) Have you had any of the following?
   1. Recent hospitalization (within 1 month, > 3 days)
   2. Recovering from recent surgery
   3. Active wound care
   4. New diagnosis with disease-related complications
   5. Start of medical treatment (hemodialysis, chemo, radiation, wound care)
   6. Recent unintentional weight loss
      - **IF NO** 🡪 **home-delivered meals (not medically-tailored)**

** Paid, low cost, and free options are needed to meet financial ability

- - - **IF YES - home-delivered medically tailored meals**

1. Do you think you will have problems physically getting food to your house in the next month?
   1. **IF YES 🡪 home-delivered groceries or food boxes**
2. Do you think you will have problems affording the food you need in the next month?
   1. **IF YES 🡪** **SNAP assistance (if don’t have already), benefits referral, food pantries**

SUPPLEMENTARY NEEDS QUESTIONS

1. Do you need basic cooking education? – Y/N
   1. **IF YES 🡪 referral for cooking education classes**
2. Do you need education to understand what healthy eating is for you? – Y/N
   1. **IF YES 🡪 referral for nutrition education**

OTHER DATA ELEMENTS TO CAPTURE

1. Are you a senior – Y/N
2. Are you on disability? – Y/N
3. Do you currently receive:
   1. SNAP – Y/N
   2. WIC – Y/N
   3. Food through a program – Y/N – If yes, name of program(s): ________________________
